# Merging Metabolism and Power: Development of a Novel Photobioelectric Device Driven by Photosynthesis and Respiration

**DOI:** 10.1371/journal.pone.0086518

**Published:** 2014-01-22

**Authors:** Ryan J. Powell, Ryan White, Russell T. Hill

**Affiliations:** 1 Institute of Marine and Environmental Technology, University of Maryland Center for Environmental Science, Baltimore, Maryland, United States of America; 2 Department of Chemistry and Biochemistry, University of Maryland Baltimore County, Baltimore, Maryland, United States of America; University of South Florida College of Medicine, United States of America

## Abstract

Generation of renewable energy is one of the grand challenges facing our society. We present a new bio-electric technology driven by chemical gradients generated by photosynthesis and respiration. The system does not require pure cultures nor particular species as it works with the core metabolic principles that define phototrophs and heterotrophs. The biology is interfaced with electrochemistry with an alkaline aluminum oxide cell design. In field trials we show the system is robust and can work with an undefined natural microbial community. Power generated is light and photosynthesis dependent. It achieved a peak power output of 33 watts/m^2^ electrode. The design is simple, low cost and works with the biological processes driving the system by removing waste products that can impede growth. This system is a new class of bio-electric device and may have practical implications for algal biofuel production and powering remote sensing devices.

## Introduction

The search for renewable energy sources has renewed interest in finding ways to use biological systems to generate electrical energy. Specifically there is an interest in systems that use biology to convert light into electrical energy as way to use the advantages of biology to harvest a sustainable energy source. These devices are collectively known as photo-bioelectric systems. In this study we aim to develop a new photo-bioelectric system through the combination of several well-known technologies and widely conserved biological phenomena. The system is distinct from a microbial fuel cell (MFC), as it uses an aluminum oxide cell design to interface phototrophic and heterotrophic metabolisms with power production.

Many photo-bioelectric systems are modeled on microbial fuel cells and have been described [1], [2]. A MFC has microbes associated with the anode oxidizing organic compounds under normally anaerobic conditions, using the anode as the terminal electron acceptor [3]. Electrons are shuttled to a platinum cathode where they combine with O_2_ and H^+^, yielding water [3]. In the closest related class of photo-MFCs, the MFC is fed organic carbon from algae [4]–[6] or plant root exudates [7]–[12]. Energy stored by photosynthesis is liberated when the organic matter is oxidized by bacteria. In another design, algae are added to the cathodic side of the MFC, supplying O_2_ in the electron consuming reaction [5]. In both cases electrons are donated from electron transfer chains to the anode. Cyanobacteria can also donate electrons to the anode during respiration of cellular carbon reserves in dark phases with redox shuttles such as HNQ [13]–[15]. The need for a redox shuttle to move electrons from algae to anode limits this type of cell to closed systems. Recently it was shown pure cultures of cyanobacteria could directly donate electrons to the anode [16]. The authors postulate these organisms donate electrons via nanowires when CO_2_ is limiting [16]. It is also possible to extract electrons from photosynthesis by using hydrogen as an intermediary [2]. Hydrogen is produced by hydrogenases or nitrogenases and then oxidized at a platinum electrode, recovering the electrons [2]. Ryu et al. take a different approach by inserting nano-electrodes directly into photosynthetic membranes of alga, extracting electrons using an overvoltage [17]. This eliminates light to chemical energy conversion losses, theoretically increasing efficiency, but consumes energy needed by the organism for growth and sustained survival.

In this study we present a system that is distinct from established photo-bioelectric systems. It is designed around the normal processes that occur when phototrophs and heterotrophs grow and replicate. Phototrophs and heterotrophs pump carbon through ecosystems, shifting inorganic carbon equilibrium reactions and in the process affecting pH. Algae alter pH by removing CO_2_ and HCO_3_^−^, which shift the equilibrium and produces hydroxide ions, resulting in pH values as high as 11 [18]. Respiration oxidizes organic compounds to CO_2_ and organic acids, reducing pH. The system extracts energy from acids and bases which are waste products of heterotrophic and phototrophic metabolism respectively. These products of metabolism are interfaced with an electrical system by using an aluminum fuel cell under the alkaline conditions. In this type of fuel cell, water and base react with aluminum to form aluminum oxides and electrons [19]. In an isolated system the electrons typically reduce hydrogen ions resulting in hydrogen gas, however they can also be drawn into an external circuit with an applied voltage.

The goal of this study is to demonstrate a proof of principle of exploiting the chemical gradient that occurs when photosynthesis and respiration are separated. The mechanistic data presented focus on the algal side of the cell. We show power production in the cell is pH dependent in the absence of algae. Power production is light and photosynthesis dependent when the algae are present. Finally a cell was deployed in the field to test the robustness of the design outside of the laboratory.

## Materials and Methods

### Algal Cultivation

*Nannochloropsis oceanica* IMET1 was grown in f/2 medium [20] with a salinity of 20 ppt. The alga was grown in photobioreactors which consisted of 500 ml borosilicate bottles with three ports in the cap. Two ports were connected to tubes leading to the bottom of the bottle. Air was pumped into these two ports through 0.22 µm syringe filters at a rate of 5 L/min to provide constant mixing and to provide CO_2_ present in the air. The third port was used as a vent. Light was provided by 215 watt Phillips white fluorescent lights with a light intensity of 275 µmol/m^2^/s at the front of the bottle. Cultures were grown with a light/dark photoperiod of 14/10 at 25°C. Algae were subcultured weekly using an inoculating volume of 10%. *Scenedesmus* sp. HTB1 was grown in a photobioreactor as described above and was grown in BG11 medium [21]. *Microcystis aeruginosa* LE-3 was grown in BG11 medium in 1 L Erlenmeyer flasks at 32°C with constant shaking. Light was provided with fluorescent lamps at an intensity of 100 µmol/m^2^/s on a 14/10 day/night cycle.

### Test Cell Design

The test cell was a sandwich type cell, whose structure was made of two end pieces of acrylic (100×90×13 mm each) and at least two sections of clear PVC pipe (88 mm outer diameter, 6 mm wall thickness). Unless otherwise noted two 30 mm long lengths of pipe composed the standard test cell. The cells were held together by compression from four bolts in the corners of the cell. Circular gaskets made of three layers of parafilm were placed between the junctions of each component to form a water-tight seal upon compression. A single circle of Whatman 3 filter paper (Frederick, MD) was used as the membrane separating the two sides of the cell. The Al electrode in the cell was derived from Al foil (98.5% Al) and was routinely 57 cm^2^. The platinum electrode was Pt wire of 0.3 mm diameter and an exposed surface area of 4.56 cm^2^. The power curve of the cell was performed with *N. oceanica* IMET1 at pH 10 on the high pH side and f/2 medium with a pH of 7 on the low pH side. Voltage was measured at set resistances between 5–10,000 Ω.

### Salt/Agar Cell Design

The salt agar cell is a molded cell produced by casting wells into agar using 400 ml serum bottles to form the wells. Molten agar with 5 M NaCl was poured into a plastic bin that contained the bottles separated by 2 cm. Once the agar solidified the bottles were removed leaving two separate wells. In one well *N. oceanica* IMET1 culture at pH 10.4 was added and into the other well ASW at pH 4 was added. An Al foil electrode was used on the high pH side with a submerged area of 252 cm^2^ and a Pt wire electrode was inserted in the low pH side of the electrode with an area of 4.26 cm^2^. A power curve was done to determine the optimal load to measure power output. Peak power output was measured at 500 ohms.

### Outdoor Cell Design

The larger outdoor cell was made of concentric cylinders with different materials composing each cylinder. The central cylinder is structural and consists of PVC pipe (8.8 cm diameter, 42 cm length) capped with two plastic circles of 26 cm in diameter. Platinum cloth (Fuelcellstore.com) was used as the inner electrode and had a bulk area of 600 cm^2^ of which 342 cm^2^ (57%) was projected Pt surface area. This electrode was wrapped around the inner 88 mm diameter pipe. A secondary support with a diameter of 21.5 cm supported the paper membrane which was two layers of black poster board sealed tightly at the top and bottom of the cell. Together these formed the inner, low pH side, of the cell with a volume of approximately 12.7 L. The Al electrode was composed of Al screen wrapped around the paper membrane. The high pH side of the cell in this case was the body of water where the cell is deployed. The power curve for the cell was performed with pH 10.3 water on the outside of the cell and pH 7.8 water on the inside of the cell. Voltage was measured at set resistances between 0.2–10,000 Ω.

### pH Dependence Experiment

The pH dependence of the cell was determined using the small test cell. In this experiment cell free media were used to allow more precise control of pH. The low pH side of the cell had pH 7 f/2 medium. The high pH side of the cell was set up as a flow-through system and was connected to a gradient maker that allowed pH to be precisely set by mixing high pH and low pH solutions of f/2 medium. The pH at the Al electrode was recorded every 60 seconds. Voltage from the cell was measured continuously across a 10 K Ω resistor using the HOBO data logger.

### Short Term Light/Dark Experiment

The smaller test cell was used for this experiment along with the circuit shown in fig. S3. To increase the light absorption area algae were continuously pumped through the cell and a tubular type photobioreactor on top of a fluorescent light box, giving the algae 400 cm^2^ of surface area to absorb light at an average intensity of 117 µmol/m^2^/s. This setup was light-proofed so only light from the light box reached the algae. Light and dark cycling was then controlled by turning the light box on and off with cycle lengths of four hours. The low pH side of the cell contained f/2 medium at pH 7. Two voltage channels were recorded on a HOBO U12 4-channel data logger (Onset Computer Corp., Bourne, MA). The first channel measures the voltage of the cell across a 10 K Ω resistor and the second channel measures pH from an Orion Research pH meter using the voltage outputs on the meter. The voltage output from the pH meter was amplified using an Op-Amp circuit shown in Fig. S3 to ensure the signal was in the sensitivity range of the data logger. Voltage data was converted to pH using a standard curve generated from voltage outputs at known pH values. The grounds within the logger are tied together. To prevent interference between the cell voltage channel and the pH voltage channel an oscillator circuit was built so only one channel was being measured at any moment while the other was disconnected. It was necessary to electronically insulate the pH probe from the electrodes in the cells to prevent spurious pH readings. This was achieved by passing the algae through two drip insulators on either side of the electrode which broke any electrical connection between the pH electrode and the electrode in the cell while allowing continuous pH monitoring. Periodic samples were tested to confirm the pH values recorded reflected the pH values at the electrode.

### Long Term Light/Dark Experiment

The smaller test cell was used for this experiment. The cell was started with pH 10.4 algae on the high pH side and pH 7 f/2 medium on the low pH side. The cell was incubated in a photoperiod room with a light/dark cycle of 14/10. The algal side of the cell was illuminated with Phillips white fluorescent lights with a light intensity of 275 µmol/m^2^/s. Voltage was measured continuously with a HOBO data logger over a 5K Ω resistor.

### Inhibition of Photosynthesis

The DCMU concentration needed to inhibit *Nannochloropsis* was determined by empirical testing to be 100 µM. This concentration was used for all experiments. Fresh 100 mM stocks of DCMU (Sigma) were dissolved in acetone prior to running each experiment. The small test cell was used and voltage was measured continuously with the HOBO data logger across a 10 K Ω resistor. Experiments were done with 2 L batches of algae in polypropylene bags placed directly on a light box to maximize light exposure. The illuminated surface area was 1288 cm^2^ with an average light intensity of 117 µmol/m^2^/s. Voltage and pH were measured from a 100 ml aliquot of algae, sampled every 30 minutes. A fresh 100 ml aliquot of f/2 media was used at each of these time point. Between time points the cell was washed twice with pH 2 dH_2_O to remove remaining algae and any Al reaction end products. DCMU was added after the midpoint of the timecourse to a final concentration of 100 µM. Acetone only was added as the control.

### Electrode Ionization

The small test cell was used in this experiment. Algae with pH 10.3 were added to the high pH side of the cell and f/2 medium with a pH of 7 was added to the low pH side of the cell. The two electrodes were connected directly with copper wire with no additional resistance to facilitate electron transfer. The cell was operated continuously for ten days and the electrode was observed.

### Field Test of Algal Electric Cell

The testing site was at latitude and longitude of 38.518462, −75.964653. The site is on public land and no samples were removed from the site. No permits were required to access the site. This site was chosen because periodic algal blooms occur where pH values can reach over pH 10, representing ideal conditions for the algal electric cell being tested. The cell was installed at fixed height on a dock. An aliquot of water from the pond was adjusted to pH 7 with 5 M HCl and used as the starting catholyte inside of the cell. Voltage was measured across a fixed resistance of 50 Ω every 30 seconds using the voltage input of the HOBO data logger mentioned above starting on 22^nd^ of August to the 5^th^ of September, 2012. Ohm’s law was used to convert resistance and voltage data to power in watts. Tidal data were collected from a local NOAA tide gauge (station ID: 8571892) in Cambridge, MD.

## Results and Discussion

**Figure 1:**
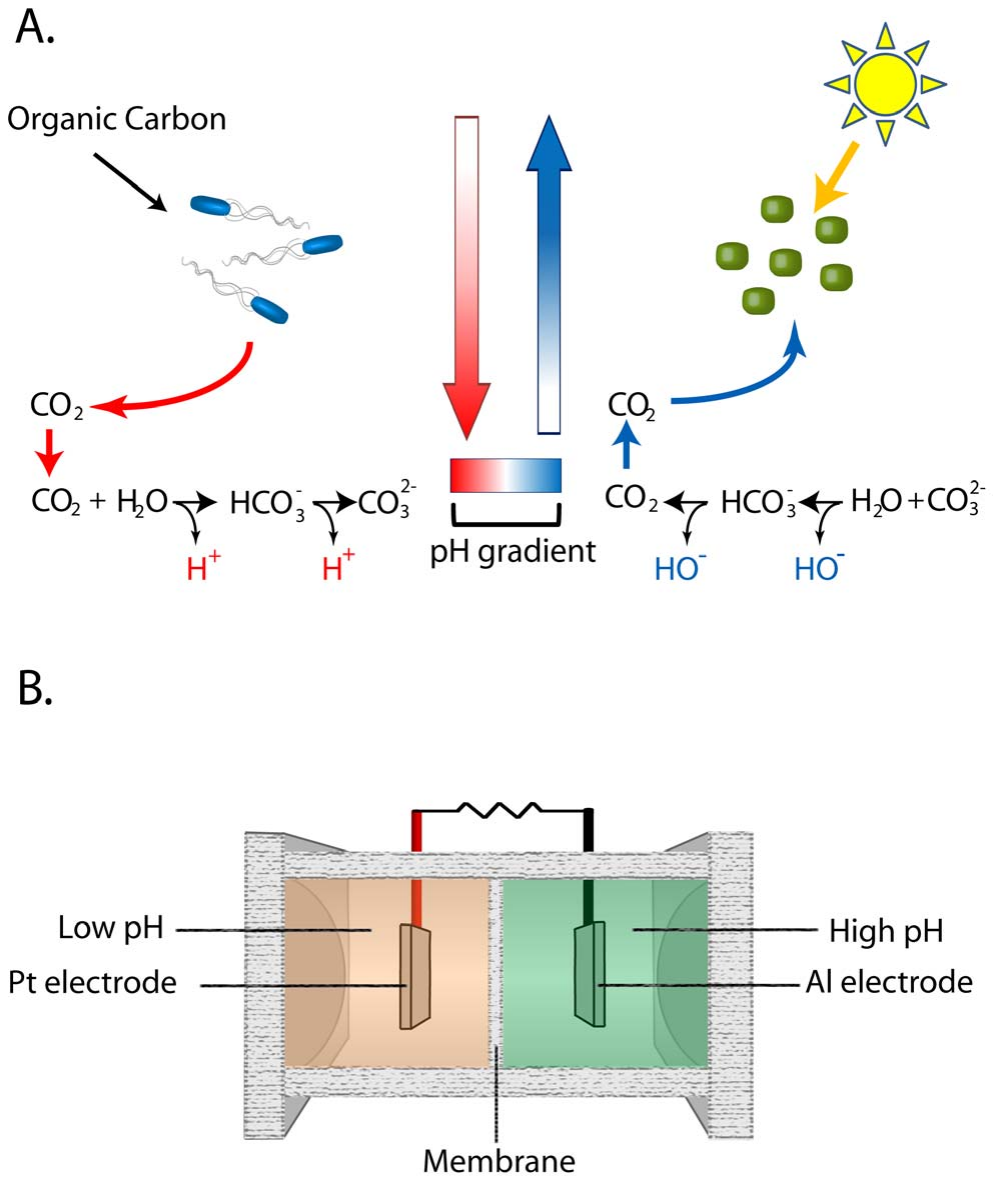
Principle and design of the algal electric cell. (**A**) Respiration decreases pH through the oxidation of organic compounds, generating CO_2_ which shifts the inorganic carbon equilibrium and produces hydrogen ions. Photosynthesis increases pH by removing CO_2_ and HCO_3_^−^, which shifts the inorganic carbon equilibrium and produces hydroxide ions, resulting in pH values as high as 11 ^23^. Separation of photosynthesis and respiration results in a pH gradient which can be used to generate electricity. (**B**) Design of test cell.

The system is shown in figure 1A with the proposed mechanism of action. By separating heterotrophic and phototrophic metabolism a pH gradient can be generated. This pH gradient can be used to generate power. Importantly, the presented system is effectively a photosynthesis dependent battery in the current stage of development due to the use of aluminum (Al) as the anode and does not yet constitute a standalone energy generation system. Substitution of the Al electrode with a catalyst would make it a stand-alone biologically driven power source. The cell has an algae-filled high pH side with an Al electrode and low pH side with a platinum electrode, separated by a paper membrane (Fig. 1B). The Al reacts with basic water produced by algal carbon fixation. Al is ionized, leaving electrons on the anode which pass through an external circuit, combining with hydrogen ions at the platinum electrode producing H_2_ (shown below).
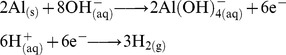



**Figure 2:**
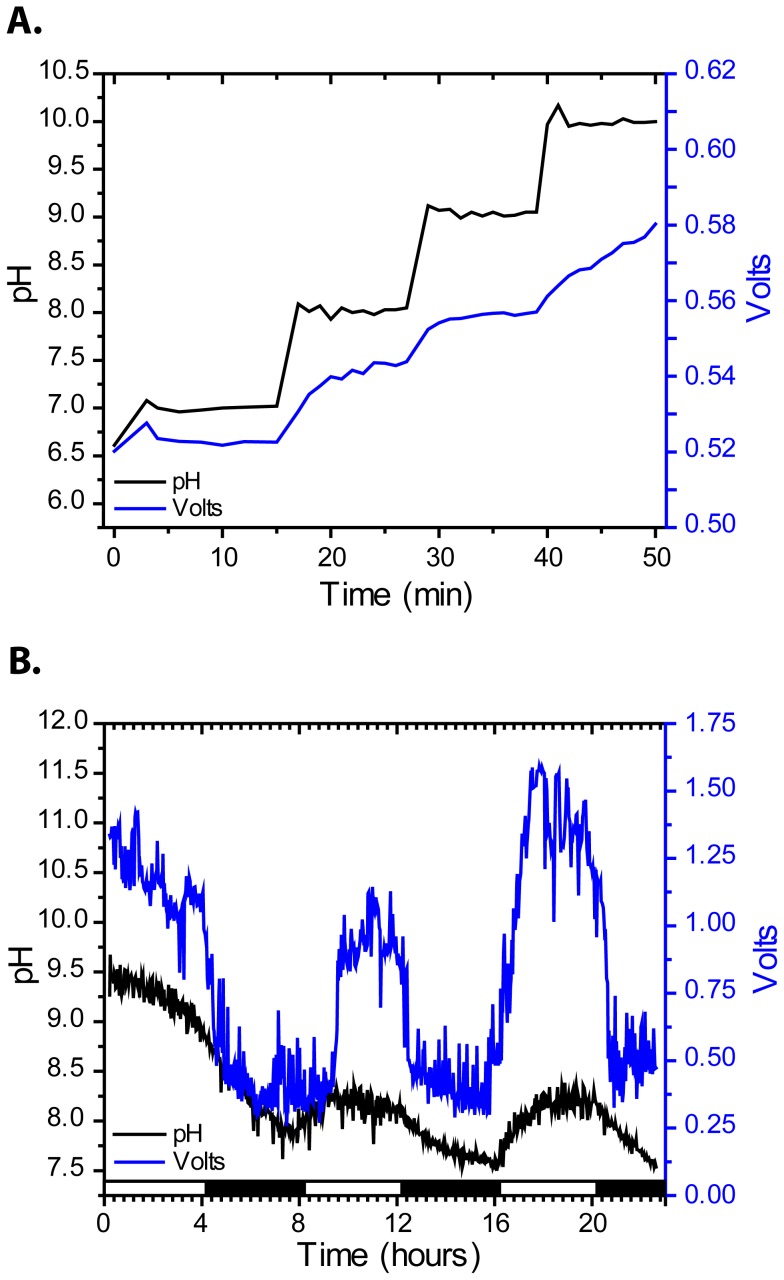
pH dependence of voltage and light dependence of pH and voltage. (**A**) Voltage is dependent on pH of the Al side of the cell. pH was adjusted stepwise in the absence of algal cells. pH was measured every minute and voltage was measured every ten seconds (**B**) Voltage and pH over light/dark cycles (white/black bar). Algal photosynthesis increases pH, increasing voltage. pH and voltage were each measured every 1.5 minutes for 24 hours. At 16 hours fresh f/2 medium was added to compensate for evaporative losses, resulting in slightly higher voltage at a lower pH due to more of the electrode being submerged. The light/dark data presented are from a single cell and are a representative dataset from four independent trials.

Initially we show the cell is responsive to pH by increasing pH stepwise on the basic side of the cell in the absence of algae while voltage was monitored. The data confirm increased pH results in increased voltage (Fig. 2A). Because voltage is dependent on having high pH on one side of the cell and low pH on the other, the greater the difference the higher the voltage as seen in Fig. 2A.

Results were obtained in cell-free media, showing living cells are not necessary for pH-dependent power production. We observed a steady-state voltage at lower pH values but at pHs higher than ca. 10, voltage continued to increase. One possibility is that the reaction has not yet achieved steady state at the surface of the aluminum at these higher pHs. Aluminum oxidation chemistry at the surface of the metal is complex and not yet completely understood, however we hypothesize that the hydroxide is responsible for the formation of an initial oxide which then reacts with water to form the metal hydroxide. One or more of these reactions may not yet have reached equilibrium.

Support for the proposed Al reaction was observed by comparing electrodes before and after ten days of operation. Electrode ionization was observed near the air-liquid interface (Fig. S1). These data support hydroxide dependent production of current through the oxidation of Al. The exact mechanism of Al oxidation in our system is unknown and may proceed by several parallel reactions [19]. While the intermediate steps in Al oxidation are important, here we focus on developing a process linking the biology of algae to an electron producing reaction.

Optimal operating load was determined with a power curve, where current was measured at increasing external resistances (Fig. S2). Optimal resistance was 500 Ω, where 285 mW/m^2^ Pt electrode was produced. Often in photo-bioelectric systems either algae or a bacterial intermediary must first colonize the electrode [2]. In our system voltage is produced immediately upon submersion of the electrodes, showing colonization is not required, which is consistent with power generation being driven by the pH. In addition, different alga in different media with similar pH values produce similar voltages including *Microcystis aeruginosa* LE-3 (freshwater cyanobacterium), *Scenedesmus* sp. HTB1 (freshwater green alga) and *Nannochloropsis oceanica* IMET1 (marine alga).

**Figure 3:**
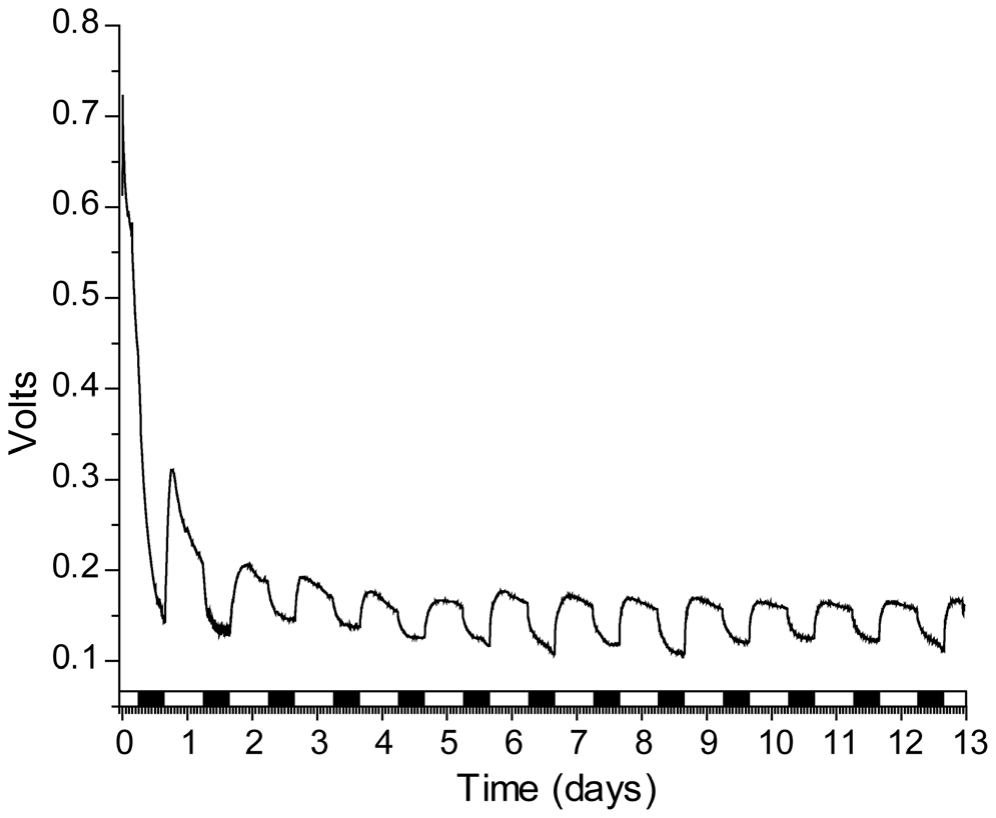
Change in voltage is light dependent. Algal electric cell with algae exposed to light (white) and dark (black) cycles for 13 days. Voltage increases when light falls on the algal electric cell, plateaus and then decreases in the dark.

**Figure 4:**
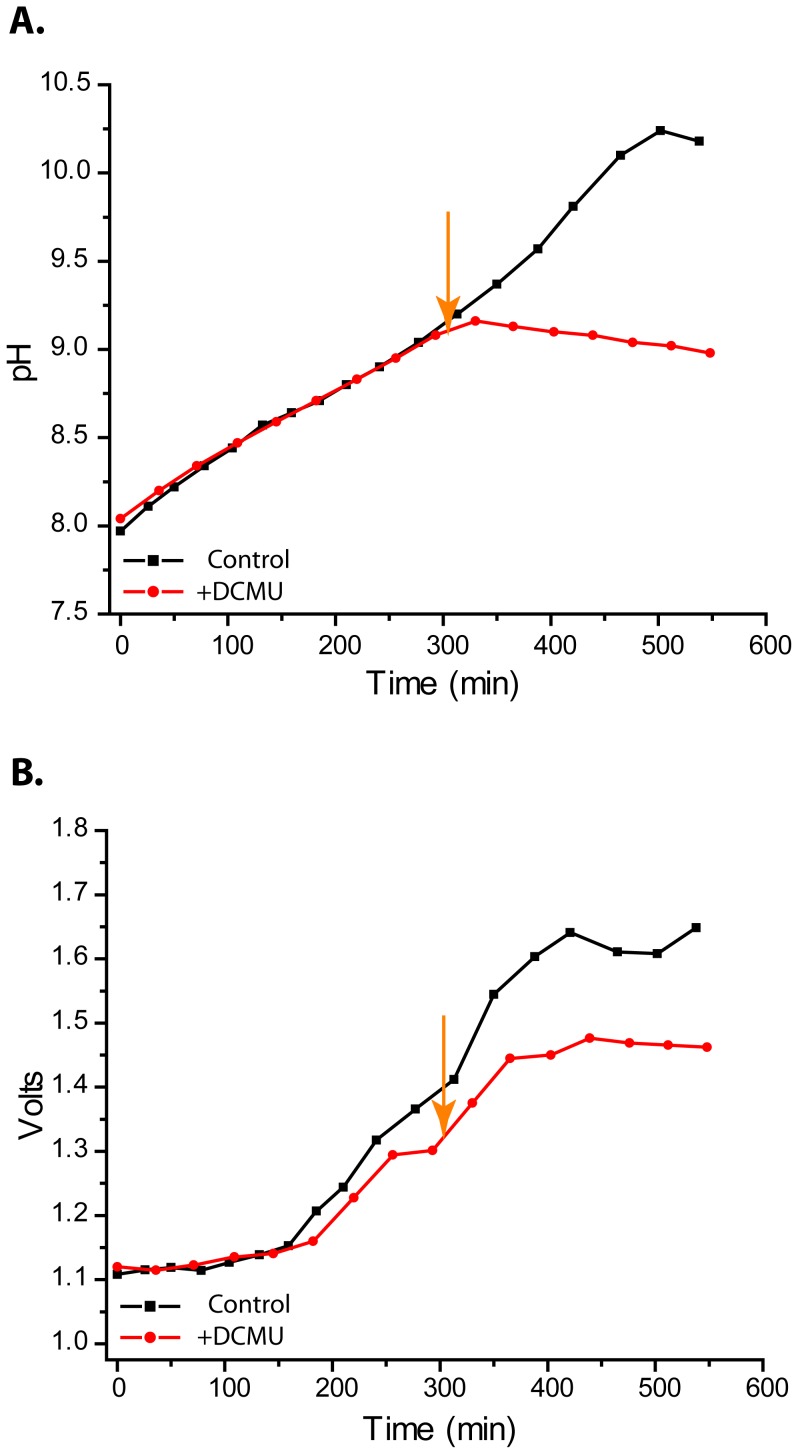
Voltage and pH are dependent on photosynthesis. Addition of the photosynthesis inhibitor DCMU (orange arrow) (**A**) inhibits pH increase, and (**B**) voltage increase. Control and inhibition experiments were carried out on separate algal stocks on separate days showing biological reproducibility of pH and voltage curves prior to addition of inhibitor. Data presented are from a single cell and are a representative dataset from three independent trials.

We hypothesized production of hydroxide ions through the removal of inorganic carbon via photosynthesis drives power production. In our next set of experiments we tested if voltage is light dependent. Algae in the cell were passed through a series of light/dark cycles while voltage and pH were monitored. During light phases, pH and voltage increase (Fig. 2B). Photosynthesis likely causes the pH increase and leads to higher cell voltage. During dark phases, photosynthesis stops and voltage decreases as hydroxide is consumed in the reaction with Al. In a separate trial the light/dark dependence was tested and found to be stable over the two week course of the experiment (Fig. 3). These data show increases in voltage are light dependent and likely due to pH increases from photosynthesis. The results also show that the same magnitude of voltage is produced when the same magnitude of pH gradient is present. To confirm power output is dependent on photosynthesis we used DCMU (3-(3,4-dichlorophenyl)-1,1-dimethylure), a specific inhibitor of photosystem II. DCMU was added to cultures under constant illumination while voltage and pH were monitored. Upon addition of the inhibitor pH increase stopped, whereas the pH of the solvent control lacking DCMU continued to climb (Fig. 4A). Cell voltage resembles the pH curve, voltage increase stopped after addition of the inhibitor, while voltage continued to climb in the control (Fig. 4B). Voltage plateaued before pH in the control, potentially indicating inhibition by constraints on the cathodic reaction imposed by the size of the Pt electrode, or the internal resistance of the cell. The data show specific inhibition of photosynthesis results in lower pH and lower voltage, confirming voltage is light and photosynthesis dependent.

Next we were interested in testing the limits of this type of cell. We hypothesized power was limited by internal resistance; this was tested with a cell cast into 1.5% agar with 5 M NaCl. This setup produced a peak power output of 33 W/m^2^ Pt electrode with a stable power output of 0.7 W/m^2^ Pt. This peak power output is over 33 fold higher than the next highest photo-bioelectric cell [6] and nearly 6 fold higher than the highest bioelectric system [22]. High power is possible due to liberation of stored energy in the Al electrode through reaction with algae-derived hydroxide. The Al electrode could be substituted with a catalyst to extract electrons from hydroxide ions such as a perovskite oxide [23]. It should also be possible to increase power by decreasing the pH of the acidic side of the cell, though the focus of the current paper is on the algal side of the cell. The current device is useful for powering remote sensors in aquatic systems and for algal biofuel production to control pH and provide power for mixing ponds.

**Figure 5:**
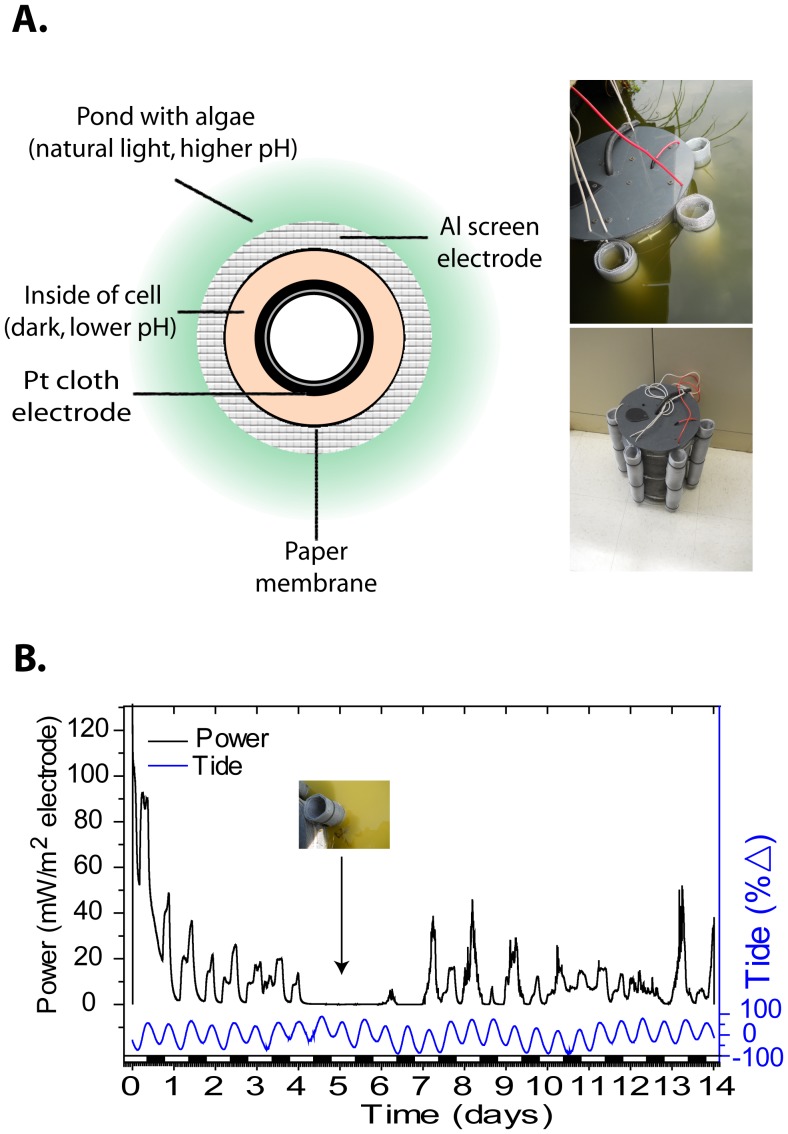
Design and field-testing of the outdoor photo-bioelectric cell. (**A**) Cutaway of the cell. Inside of the cell is water with the local biota which is kept in the dark, inhibiting photosynthesis while allowing respiration. Pt cloth electrode is wrapped around the center. Paper membrane separates the inside of the cell from the surrounding water. Al electrode is wrapped around the cell. (**B**) Power produced by the cell. Power is correlated with day/night cycle (white/black bar) and tides (blue). After day four a storm suspended sediment, likely inhibiting photosynthesis and power production. Voltage was measured every 30 seconds for two weeks over a 50 Ω resistance. Data presented are from a single cell deployed on-site.

The mechanism of action suggests the cell is species neutral. The species neutral nature of the cell was tested with a larger version deployed in a Chesapeake Bay tributary. This cell incorporated the entire proposed design principle and is shown in figure 5A. A power curve showed peak power output occurred at 50 Ω resistance (Fig. S2). Power was produced immediately upon submerging the cell at the site, where pH was 8.3. The cell interior maintained a lower pH than the surrounding water throughout the experiment. A maximum power output of 192 mW/m^2^ Pt electrode was recorded shortly after start up with an average power output of 11 mW/m^2^ Pt (Fig. 5B). Unlike the laboratory-scale test, the cell was affected by environmental factors including storms and tides. Power produced roughly follows the day/night cycle, deviations from this cycle correlate well with changes in tides. Changes in tides affect power output by altering the area of submerged Pt electrode. After the fourth day a storm moved over the site and stirred up sediment, correlating with a drop in power to 0.3–1 mW/m^2^ Pt electrode (Fig. 5B). We speculate this was due to a pH decrease from inhibition of photosynthesis. After this period the cell recovered, and resumed power production. From these data we conclude the cell can successfully produces power with an undefined freshwater microbial community. The system we presented represents a novel class of photo-bioelectric cell, where photosynthesis and respiration are linked to electrical power production via known electrochemical reactions. This type of cell can generate high current densities and operate outside of the laboratory in a species neutral manner. Power production from different species is likely dependent on the rate at which algae removed inorganic carbon from water. This rate will differ based on the algal species present and the densities on those algae. To maintain a constant voltage from the system under differing conditions of algal carbon fixation the external resistance can be adjusted to obtain a desired voltage.

It is important to remember that the current cell design is dependent on oxidation of aluminum and therefore does not yet constitute a stand-alone sustainable energy source. It may however be used as a way to liberate the energy stored in the reduced metal in a portable manner. This would allow aluminum to be used effectively as an energy storage medium for transferring energy from areas that have access to large reserves of renewable energy, such as hydroelectric power, to areas that do not. Further research may allow power to be generated directly from the pH gradient that occurs when photosynthetic carbon fixation is separated from respiration.

## Supporting Information

Figure S1 **Ionization of the Al electrode, demonstrating solid Al metal is being dissolved.** On the left is the original electrode and on the right is the same electrode after 10 days of operation. The electrode on the right has completely dissolved near the liquid interface and has small holes in the submerged portion of the electrode.
(TIF)

Figure S2 **Power curves for the small test cell and the large outdoor cell. (A)** Power curve for the outdoor cell. **(B)** Rescaled view of the outdoor cell’s power curve showing peak power output at 50 Ω resistance. **(C)** Power curve for small test cell. **(D)** Rescaled view of the small test cell’s power curve showing peak power output at 500 Ω resistance.
(TIF)

Figure S3 **Diagram of the circuit used to measure pH and voltage continuously.** Common grounding within the data logger requires the pH channel and the algal cell voltage channel to be alternately measured so they do not interfere with the measurement. In this circuit two 9 V sources run through an oscillator which alternately connects and disconnects each 9 V source for 30 seconds. When connected, the current activates an NPN transistor, which acts as an electronic switch, allowing the voltage from the cell to continue on to the data logger shown to the right. For pH measurement the transistor gates a low voltage output from the pH meter. This output continues on to an Op-Amp where the signal is amplified to be within the optimal voltage range of the data logger. Circuit diagram was drawn in circuitlab (www.circuitlab.com).
(TIF)

## References

[1] Strik DPBTB, Timmers RA, Helder M, Steinbusch KJJ, Hamelers HVM, et al (2011) Microbial solar cells: applying photosynthetic and electrochemically active organisms. Trends in Biotechnology 29: 41–49. 21067833 10.1016/j.tibtech.2010.10.001

[2] Rosenbaum M, He Z, Angenent LT (2010) Light energy to bioelectricity: photosynthetic microbial fuel cells. Current Opinion in Biotechnology 21: 259–264. 20378333 10.1016/j.copbio.2010.03.010

[3] Rabaey K, Verstraete W (2005) Microbial fuel cells: novel biotechnology for energy generation. Trends in Biotechnology 23: 291–298. 15922081 10.1016/j.tibtech.2005.04.008

[4] Strik DP, Terlouw H, Hamelers HV, Buisman CJ (2008) Renewable sustainable biocatalyzed electricity production in a photosynthetic algal microbial fuel cell (PAMFC). Appl Microbiol Biotechnol 81: 659–668. 18797867 10.1007/s00253-008-1679-8

[5] De Schamphelaire L, Verstraete W (2009) Revival of the biological sunlight-to-biogas energy conversion system. Biotechnol Bioeng 103: 296–304. 19180645 10.1002/bit.22257

[6] Velasquez-Orta SB, Curtis TP, Logan BE (2009) Energy From Algae Using Microbial Fuel Cells. Biotechnology and Bioengineering 103: 1068–1076. 19418564 10.1002/bit.22346

[7] De Schamphelaire L, Van den Bossche L, Dang HS, Hofte M, Boon N, et al (2008) Microbial fuel cells generating electricity from rhizodeposits of rice plants. Environ Sci Technol 42: 3053–3058. 18497165 10.1021/es071938w

[8] Kaku N, Yonezawa N, Kodama Y, Watanabe K (2008) Plant/microbe cooperation for electricity generation in a rice paddy field. Appl Microbiol Biotechnol 79: 43–49. 18320186 10.1007/s00253-008-1410-9

[9] Timmers RA, Strik DP, Hamelers HV, Buisman CJ (2010) Long-term performance of a plant microbial fuel cell with *Spartina anglica.* . Appl Microbiol Biotechnol 86: 973–981. 20127236 10.1007/s00253-010-2440-7 PMC2841269

[10] Helder M, Strik D, Hamelers H, Kuhn A, Blok C, et al (2010) Concurrent bio-electricity and biomass production in three Plant-Microbial Fuel Cells using *Spartina anglica*, *Arundinella anomala* and *Arundo donax.* . Bioresource technology 101: 3541–3547. 20097554 10.1016/j.biortech.2009.12.124

[11] Takanezawa K, Nishio K, Kato S, Hashimoto K, Watanabe K (2010) Factors affecting electric output from rice-paddy microbial fuel cells. Bioscience, biotechnology, and biochemistry 74: 1271–1273. 10.1271/bbb.90852 20530890

[12] De Schamphelaire L, Cabezas A, Marzorati M, Friedrich MW, Boon N, et al (2010) Microbial community analysis of anodes from sediment microbial fuel cells powered by rhizodeposits of living rice plants. Applied and environmental microbiology 76: 2002–2008. 20097806 10.1128/AEM.02432-09 PMC2838000

[13] Tanaka K, Tamamushi R, Ogawa T (2009) Bioelectrochemical fuel-cells operated by the cyanobacterium, *Anabaena variabilis* . Journal of Chemical Technology and Biotechnology Biotechnology 35: 191–197.

[14] Yagishita T, Sawayama S, Tsukahara KI, Ogi T (1998) Performance of photosynthetic electrochemical cells using immobilized *Anabaena variabilis* M-3 in discharge/culture cycles. Journal of Fermentation and Bioengineering 85: 546–549.

[15] Yagishita T, Sawayama S, Tsukahara K, Ogi T (1997) Behavior of glucose degradation in *Synechocystis* sp. M-203 in bioelectrochemical fuel cells. Bioelectrochemistry and Bioenergetics 43: 177–180.

[16] Pisciotta JM, Zou Y, Baskakov IV (2010) Light-dependent electrogenic activity of cyanobacteria. PLoS One 5: e10821. 20520829 10.1371/journal.pone.0010821 PMC2876029

[17] Ryu W, Bai SJ, Park JS, Huang Z, Moseley J, et al (2010) Direct extraction of photosynthetic electrons from single algal cells by nanoprobing system. Nano Lett 10: 1137–1143. 20201533 10.1021/nl903141j

[18] Dubinsky Z, Rotem J (1974) Relations between algal populations and the pH of their media. Oecologia 16: 53–60. 28308951 10.1007/BF00345087

[19] (2008) Reaction of Aluminum with Water to Produce Hydrogen. U.S. Department of Energy.

[20] Guillard RRL (1975) Culture of phytoplankton for feeding marine invertebrates. Culture of marine invertebrate animals Plenum: 29–60.

[21] Stanier RY, Kunisawa R, Mandel M, Cohen-Bazire G (1971) Purification and properties of unicellular blue-green algae (order *Chroococcales*). Bacteriol Rev 35: 171–205. 4998365 10.1128/br.35.2.171-205.1971 PMC378380

[22] Cusick RD, Kim Y, Logan BE (2012) Energy Capture from Thermolytic Solutions in Microbial Reverse-Electrodialysis Cells. Science 335: 1474–1477. 22383807 10.1126/science.1219330

[23] Suntivich J, May KJ, Gasteiger HA, Goodenough JB, Shao-Horn Y (2011) A perovskite oxide optimized for oxygen evolution catalysis from molecular orbital principles. Science 334: 1383–1385. 22033519 10.1126/science.1212858

