# Supplementary figures and images for: Merging Metabolism and Power: Development of a Novel Photobioelectric Device Driven by Photosynthesis and Respiration
Source: PLoS One. 2014 Jan 22;9(1):e86518. doi: 10.1371/journal.pone.0086518 (PMC3899268; doi:10.1371/journal.pone.0086518)

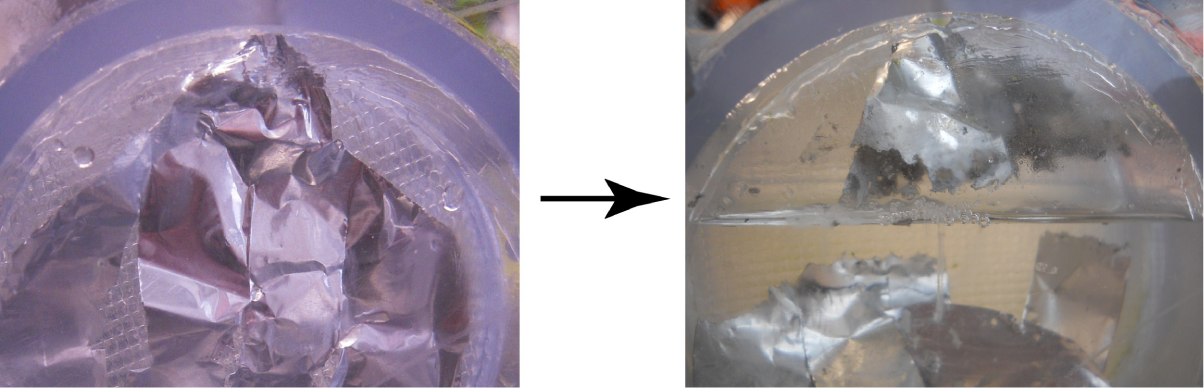

Supplement: Figure S1 — Ionization of the Al electrode, demonstrating solid Al metal is being dissolved. On the left is the original electrode and on the right is the same electrode after 10 days of operation. The electrode on the right has completely dissolved near the liquid interface and has small holes in the submerged portion of the electrode. (TIF) [file pone.0086518.s001.tif]

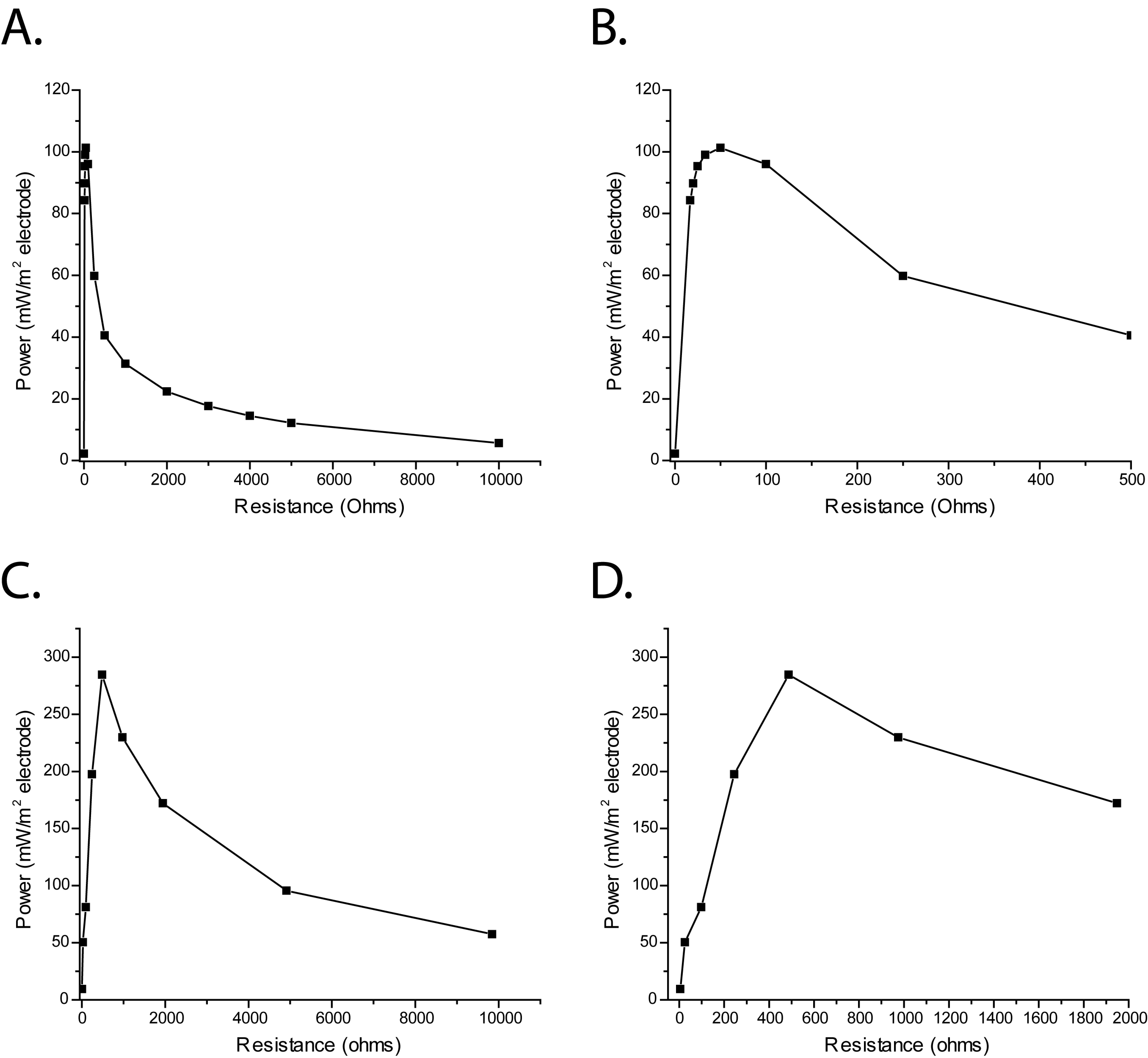

Supplement: Figure S2 — Power curves for the small test cell and the large outdoor cell. (A) Power curve for the outdoor cell. (B) Rescaled view of the outdoor cell’s power curve showing peak power output at 50 Ω resistance. (C) Power curve for small test cell. (D) Rescaled view of the small test cell’s power curve showing peak power output at 500 Ω resistance. (TIF) [file pone.0086518.s002.tif]

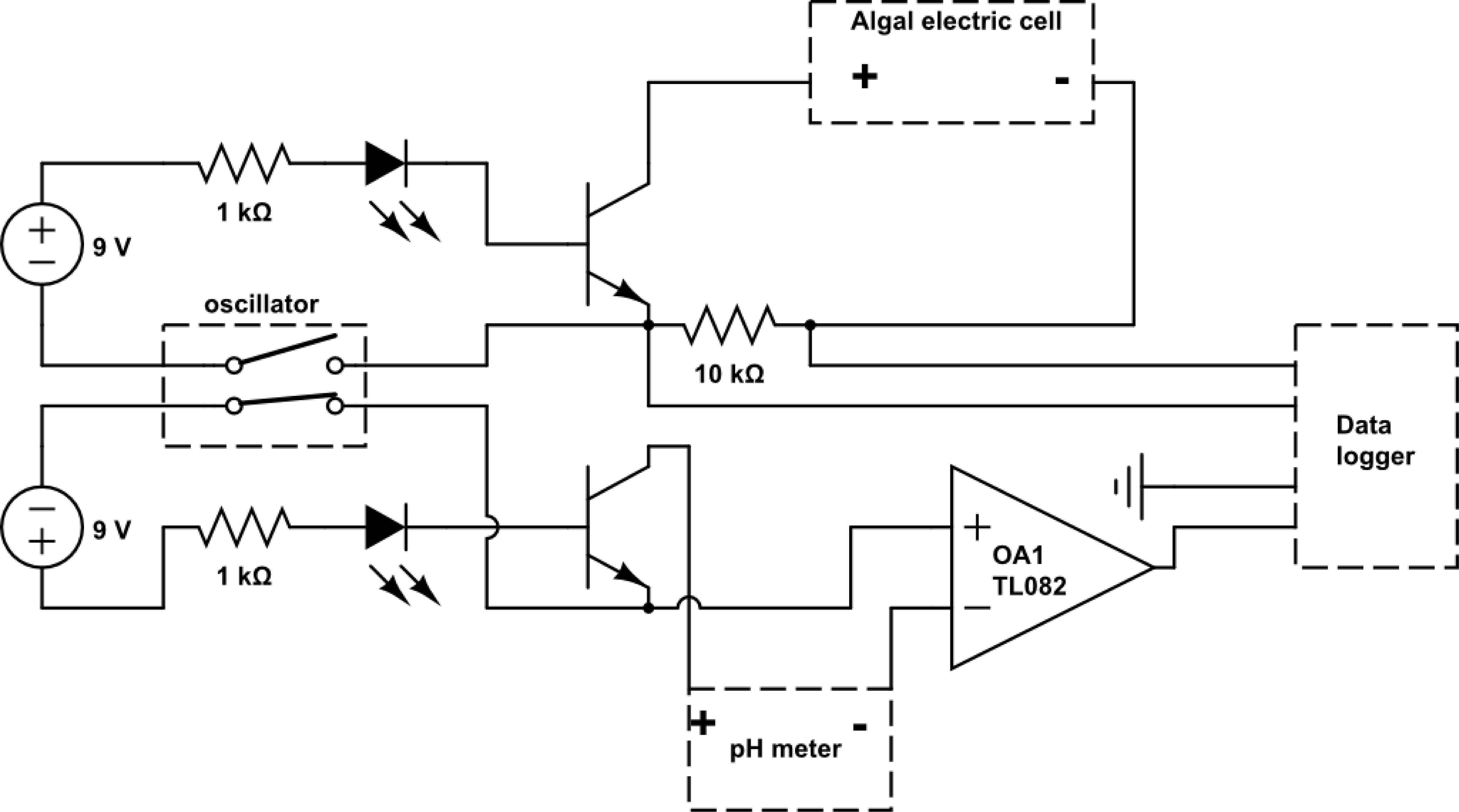

Supplement: Figure S3 — Diagram of the circuit used to measure pH and voltage continuously. Common grounding within the data logger requires the pH channel and the algal cell voltage channel to be alternately measured so they do not interfere with the measurement. In this circuit two 9 V sources run through an oscillator which alternately connects and disconnects each 9 V source for 30 seconds. When connected, the current activates an NPN transistor, which acts as an electronic switch, allowing the voltage from the cell to continue on to the data logger shown to the right. For pH measurement the transistor gates a low voltage output from the pH meter. This output continues on to an Op-Amp where the signal is amplified to be within the optimal voltage range of the data logger. Circuit diagram was drawn in circuitlab (www.circuitlab.com). (TIF) [file pone.0086518.s003.tif]
